# ESHRE certification of ART centres for good laboratory and clinical practice ^†^

**DOI:** 10.1093/hropen/hoac040

**Published:** 2022-09-14

**Authors:** Luca Gianaroli, Anna Veiga, Stephan Gordts, Thomas Ebner, Bryan Woodward, Catherine Plas, Wil van Groesen, Serena Sgargi, Borut Kovačič

**Affiliations:** SISMER Reproductive Medicine Unit, Bologna, Italy; Reproductive Medicine Service, Dexeus Mujer, Hospital Universitari Dexeus/Institut d'Investigació Biomèdica de Bellvitge, IDIBELL, Barcelona Stem Cell Bank, Regenerative Medicine Programme, Barcelona, Spain; Life Expert Centre, Leuven, Belgium; Department of Gynecology, Obstetrics, and Gynecological Endocrinology, Kepler Universitätsklinikum, Linz, Austria; X&Y Fertility, Leicester, UK; ESHRE Central Office, Strombeek-Bever, Belgium; WiDiDi BV, Zwijndrecht, Netherlands; SISMER Reproductive Medicine Unit, Bologna, Italy; Department of Reproductive Medicine and Gynecological Endocrinology, University Medical Centre Maribor, Maribor, Slovenia

**Keywords:** ART, certification, ESHRE, good clinical practice, good laboratory practice

## Abstract

**STUDY QUESTION:**

Three years after the start of the ESHRE ART Centre Certification (ARTCC) programme, what is the current state of the system, in terms of the interest expressed in it and experiences during assessment of ART services?

**SUMMARY ANSWER:**

As of 1^st^ December 2021, 25 European ART centres have been involved in the various stages of certification and the most common recommendations from inspectors were the need for documented training, verification of competencies for all staff members, verification of laboratory and clinical performance indicators, implementation of a quality management system and avoidance of overusing ICSI and add-ons.

**WHAT IS KNOWN ALREADY:**

European Union (EU) legislation has included ART activities in the EU Tissue and Cells Directives (EUTCDs). Following inspections by national EUTCD authorities, many details regarding documentation, laboratory environment, handling of reproductive cells and tissues, traceability, coding and patient testing have become standardized. However, the EUTCDs do not cover all ART-specific aspects. For this reason, the ARTCC was established to focus on peculiar areas, including relevant staff qualifications, training, continuing professional development, workload, equipment suitability, (non)-evidence-based laboratory and clinical methods used, treatment approaches according to ESHRE guidelines, recommendations and laboratory and clinical key performance indicators.

**STUDY DESIGN, SIZE, DURATION:**

The paper reviews the state of the art of the ESHRE certification of ART centres for good clinical and laboratory practice over an initial 3-year period of operation, including the number of ART centres involved in the different stages of certification and the most common recommendations by inspectors.

**PARTICIPANTS/MATERIALS, SETTING, METHODS:**

In 2016, the ARTCC working group began to establish a new ESHRE ARTCC programme. Since then, the working group has organised four preparatory courses and appointed 37 inspectors (19 clinicians, 17 embryologists and one paramedical). A tool to verify compliance with ESHRE recommendations for good laboratory and clinical practice was developed. The ARTCC has been open for applications since September 2018. In Step 1, the applicant enters basic information about the ART centre, staff and ART activities into the application platform. After review and approval, the applicant is given the opportunity to enter Step 2 and provide detailed online checklists on general, laboratory, clinical services and clinical outcomes. Two inspectors (one clinician and one embryologist) independently evaluate the submitted checklists. The condition to proceed to evaluation is a positive mean score (at least 66%) from each of the four checklists. In Step 3, a live site visit [or virtual owing to the coronavirus disease 2019 (COVID-19) pandemic] is organised and the inspectors prepare a final report with appropriate recommendations. The application may be rejected at any time if the criteria required to advance to the next stage are not met. The ARTCC programme is currently available for European countries listed in ESHRE internal rules, available on the ESHRE website. The certificate is valid for 3 years, after which an application for renewal can be submitted.

**MAIN RESULTS AND THE ROLE OF CHANCE:**

Over a 3-year period (until 1st December 2021), 63 ART centres from 25 countries started applying through an online platform. So far, 38 applications did not progress owing to lack of completion of the initial application within a 1-year period or because applications came from non-European countries. Of the remaining 25 applications, eight centres have been inspected and seven centres have been certified. The most common recommendations given by inspectors to assessed centres were the need for documented training, verification of competencies, skills and continuing professional development for all staff members, verification of laboratory and clinical performance indicators and implementation of a quality management system. The inspectors identified some recurring areas of medically assisted reproduction that deviate from good practice: the overuse of ICSI, preimplantation genetic testing for aneuploidies, freeze-all and other add-ons. They often reported that the clinical outcomes could not be objectively assessed because of non-inclusion of the started cycles or the frequent use of freeze-all cycles.

**LIMITATIONS, REASONS FOR CAUTION:**

No major modifications have been made to the application platform and checklists since the early stages of the certification programme. However, in this short time, quite a few changes in clinical practice have occurred, especially concerning the more frequent use of the “freeze-all” strategy. As a result, problems arose in the evaluation of clinical outcomes. In addition, because of the COVID-19 pandemic, site visits were substituted by the implementation of virtual visits. While this enabled the certification programme to continue, it is possible that certain critical details that would have been noticed during a traditional site visit may have been overlooked.

**WIDER IMPLICATIONS OF THE FINDINGS:**

Regular monitoring of the observations of ARTCC inspectors and analysis of their reports is certainly useful to harmonise inspectors’ criteria in the assessment process and to identify chronic deficiencies in clinical and laboratory practice. Nonconformities can be addressed by ESHRE through guidelines and recommendations, as well as through discussion with EU institutions and competent authorities.

**STUDY FUNDING/COMPETING INTEREST(S):**

The ARTCC programme was developed and funded by ESHRE, covering expenses associated with the meetings. The Steering Committee members who are the authors of this paper did not receive payments for the completion of this study. The inspectors were remunerated for their work with an honorarium. The authors have no conflicts of interest to declare.

## Introduction

Medically assisted reproduction (MAR) is a fast-growing branch of medicine. Improvements of new products (such as increasing the biological purity of fertility drugs and adapting the composition of the culture media to the needs of the embryo) are constantly increasing the safety and effectiveness of fertility treatments owing to increasingly stringent standards regulating their production and usage (e.g. the EU Medical Devices Directive), although quality standards, as well as regulations, may significantly differ among countries.

Those MAR centres where infertility is also treated with ART (ART centres) are subject to a continuous performance monitoring based on clinical and laboratory key performance indicators (KPIs). Monitoring is carried out both internally (by staff members of the centre) and externally at a national and international level (by competent authorities, registries, etc.) (de Mouzon et al., 2019).

While pregnancy rate was considered as one of the primary KPIs for evaluating the quality of ART programmes (Crawford et al., 2019), it has been accepted that ART centres may experience fluctuations in clinical outcomes, since the success of ART is influenced by numerous factors, including qualifications and training of clinical and laboratory staff (Calhaz-Jorge et al., 2015; Kovačič et al., 2015).

In the attempt to minimise the impact of these variables, many of which can be difficult to detect in real time, ART centres have developed their own quality management systems and resorted to International Organization for Standardization (ISO) certification, accreditation or other available laboratory standards (Sjöblom, 2012). The implementation of Standard Operating Procedures (SOPs), aimed at standardising work and monitoring compliance of processes with pre-defined criteria, has become a key element of quality control management (Mortimer and Mortimer, 2005).

According to European Union (EU) legislation, MAR/ART activities are included in the framework of the EU Tissue and Cells Directives (EUTCDs), for which Quality Management (QM) is only one of the mandatory requirements (European Parliament and the Council, 2004). This has largely replaced the need for ISO standardization as many details regarding documentation, laboratory environment, handling of reproductive cells and tissues, traceability, coding and patient testing have been standardised following inspections by national EUTCD competent authorities (European Directorate for the Quality of Medicines & Health Care of the Council of Europe, 2019).

However, the EUTCDs do not cover all MAR/ART-specific aspects and they do not apply to countries outside the EU. Whenever regulatory standards are not in place, professional recommendations and guidelines should be used as reference by professionals. Altogether, regulations, standards, guidelines and recommendations represent good laboratory and clinical practice in the treatment of infertility, which is of utmost importance to ensure the safety and quality of all procedures performed for both patients and staff members.

Since its foundation, ESHRE has promoted the improvement in clinical practice, the organisation of teaching and training activities and the provision of guidance to enhance safety and quality assurance in clinical and laboratory procedures. To achieve these goals, ESHRE certification programmes have been developed to accredit knowledge and skills of MAR/ART professionals.

Following the success of certification programmes for professionals (ESHRE certification of Clinical Embryologists, Nurses and Midwives and Reproductive Endoscopy Surgeons), ESHRE decided to develop a unique certification programme for ART centres based on a careful evaluation of clearly defined criteria performed by specifically trained inspectors/assessors.

The ESHRE ART Centre Certification for good clinical practice (ARTCC) was designed to focus on the assessment of the following critical areas: staff qualifications, training, continuing professional development and workload (Calhaz-Jorge et al., 2015; Kovačič et al., 2015; 2020), laboratory equipment and culture media suitability (Lundin and Ahlström, 2021; Sunde et al., 2016), techniques performed (including add-ons) (Harper et al., 2012; Provoost et al., 2014; Repping, 2019; Zhang et al., 2020; Jans et al., 2020; Nardo and Chouliaras, 2020; Human Fertilisation & Embryology Authority, 2019, www.hfea.gov.uk/treatments/treatment-add-ons/), laboratory standards (ESHRE Guideline Group on Good Practice in IVF Labs, 2016; WHO, 2021), treatment approaches according to ESHRE guidelines (Dunselman et al., 2014; European Society for Human Reproduction and Embryology (ESHRE) Guideline Group on POI, 2016; ESHRE Guideline Group on RPL, 2018; ESHRE Guideline Group on Female Fertility Preservation, 2020; Ovarian Stimulation TEGGO, 2020; ESHRE Working Group on Reproductive Donation, 2022), compliance with ESHRE recommendations (ESHRE PGT Consortium Steering Committee, 2020; ESHRE Working group on Time-lapse technology, 2020; ESHRE Working group on Chromosomal Mosaicism, in press), data management (De Geyter et al., 2020), and laboratory (ESHRE Special Interest Group of Embryology and Alpha Scientists in Reproductive Medicine, 2017) and clinical (ESHRE Clinic PI Working Group, 2021) KPIs.

The ARTCC was officially launched during the 34$^{th}$ ESHRE Annual Meeting in 2018 and in September 2018 the link to the application platform was made available on the ESHRE website for centres located in Europe. Three years after the start of the ARTCC programme, the Steering Committee presents the functioning of the system, the experience so far and the current state of the art of the certification of ART centres. The experiences, findings and recommendations of the inspectors described so far should be of benefit to both applicants and inspectors in the future.

## Materials and Methods

*Steering Committee and programme set-up*

The ARTCC Steering Committee was appointed in September 2016 to set up a system to verify compliance of ART centres with indications provided in ESHRE papers, guidelines and recommendations for the benefit of both operators and patients. In June 2017, the Steering Committee's preliminary proposal included a flow chart summarising the functioning of the programme, a draft of checklists with the related scoring system and a tentative budget. Following approval from the ESHRE Executive Committee, the ARTCC Steering Committee finalized the detailed checklists (General services: 59 questions, Laboratory services: 110 questions, Clinical services: 86 questions, and Clinical results) and an electronic platform for application management was developed.

The next step towards full implementation was the appointment of inspectors. In the second half of February 2018, ESHRE launched the first call for inspectors among its European members. Applications were reviewed by the Steering Committee and 29 (15 clinicians, 13 embryologists, one paramedical) were approved A compulsory training course for approved applicants was held in June 2018.

The Certification programme was officially presented in 2018 during the 34th ESHRE Annual Meeting and in September 2018 the link to the certification platform was made available on ESHRE website for ART centres located in Europe. Based on the number of applications received, a second call for inspectors was launched in 2019 and 15 further applications were approved (five clinicians, 10 embryologists). The second training course was held in April 2019.

Since becoming fully operational, the activities and duties of the ARTCC Steering Committee have come to include supervision of a centre's application, appointment of inspectors for review of applications and on-site visits, supervision of inspectors' activities, review of final reports, contact with the Executive Committee for periodical reporting, final reports approval, inspectors' selection and complaints management.

*Application process*

The application process for ESHRE ART Centre Certification for good clinical practice is summarized in Fig. 1A. The process consists of seven steps, each subject to verification. Figure 1B shows the certificate renewal process after 3 years.

The first part of the application form needs to be filled in online and covers general information about the centre, including authorizations, quality certifications, treatment reimbursement policies, facilities, information about the team (team composition and professional profiles of key staff members) and relevant supporting documents (licence, quality certificates, list of SOPs and consent forms). These documents have to be translated to English. Centres are also asked to indicate treatments available and for each of them provide the number of procedures performed during the last calendar year.

Once the applicant informs the ARTCC Steering Committee that the application has been completed, it enters the second step. Initial applications are subsequently reviewed by the Steering Committee to verify whether a centre complies with basic criteria in terms of licence, facilities, adequate staff composition and treatments performed.

If the initial application is approved by the Steering Committee, the applicant centre is asked to pay the first instalment of the application fee in order to obtain access to the third step of the application process. This step involves compiling four checklists (general services, laboratory services, clinical services and a table of treatments and clinical outcomes) through the ARTCC platform. Once completed, the Steering Committee appoints two inspectors (one clinician and one embryologist) to independently score the checklists. Each inspector has a scale from 0 to 100 in the platform, on which they independently rate each area of the checklists. As a key element of clinical practice review, the ARTCC Steering Committee decided to compare clinical results provided by ART centres with respective national results published by the ESHRE EIM Consortium (The European IVF-Monitoring Consortium (EIM) for the European Society of Human Reproduction and Embryology (ESHRE) et al., 2017). At least 20 procedures had to be performed per year for an evidence-based treatment to be certified. The scoring system is summarized in Supplementary Fig. S1. If the overall average score of each checklist is satisfactory (≥ 66%), inspectors arrange a site visit (usually for 2 days) at the applicant centre. The site visit is scheduled following the payment of the second instalment of the application fee.

During the site visit, inspectors check the accuracy of answers to the questions in the checklists and assess the reliability of clinical results provided using a traffic-light tool (Supplementary Fig. S2). If necessary, the preliminary checklist scoring can be updated and adjusted based on the outcome of the site visit. The weights of the individual domains from the four checklists, which each contribute to 25% of the final score, are presented in Supplementary Fig. S1.

Any deviations from good clinical practice are recorded by inspectors in the platform and recommendations for solving them are provided in the final report. If required, the Steering Committee can ask for additional documents, clarifications, or even for an extra site visit.

Scores are classified as follows: excellent (91% - 100%), good 76% - 90%), fair (66% - 75%), insufficient (<66%). If a centre receives a score that falls into the fair or insufficient category for a particular area, it does not mean that the centre cannot be certified. It may be a critical deficiency that needs to be addressed. If the score for each checklist (general services, laboratory services, clinical services and treatment outcomes) is satisfactory (≥66%) and if there are no particularly critical issues, the report is approved by the Steering Committee and sent to the Executive Committee for ratification and release of the ESHRE certificate for good clinical practice. Certification is valid for a period of 3 years. If the score does not meet the threshold level, the centre is given the opportunity to solve critical issues and re-submit the application within 1 year from recommendations, without having to pay an additional application fee. ESHRE certified ART centres are listed on the ESHRE website.

*Analysis of applications and assessment of checklists and treatment outcomes*

To evaluate the functioning of the programme 3 years after its launch, the ARTCC Steering Committee performed an analysis of applications processed as of the 1st December 2021 to check how many centres applied or showed interest in the certification, in which European countries they were located, and which were the most common issues and recommendations arising from checklist assessment and site visits. Any centre could start an application through the platform, but access to checklists was allowed only to those who successfully completed the initial application and paid the first instalment of the application fee. Data about applications and checklists scoring were collected from the online platform and from final reports ratified by the ESHRE Executive Committee.

At the beginning of 2020, with several applications at different stages of the certification programme, the COVID-19 pandemic seriously threatened its functioning. To avoid stopping all activities, the Steering Committee put in place in record time an innovative and efficient virtual site visit system. Two virtual courses were organised to update the inspectors on its functioning. Virtual site visits turned out to be an effective tool to obtain a comprehensive overview of clinical and laboratory activities performed at ART centres whenever a live site visit was not possible owing to force majeure. In addition, they might play a key role in expanding the ARTCC programme outside of Europe. The difference was in the total time inspectors had to check. For a site visit, this was 1.5 days, while for a virtual visit it was 6 hours. However, centres were asked to record individual procedures and send films to inspectors in advance.

So far, reports have been approved for eight ART centres. The scores and recommendations included in these final reports for each checklist have been included in this analysis. The most frequent non-conformities and recommendations reported by inspectors have also been summarized (Supplementary Tables SI-SIV).

Scoring categories for each checklist's subsection achieved by certified ART centres are summarized graphically in Fig. 2. For each subsection, average, minimum and maximum scores have been calculated.

*Statistical analysis*

The obtained data on interest and progress of applications are shown in the table by European countries from which the applications came. Inspectors' scores of individual parts of checklists were expressed as percentages. These assessments were then categorized into four categories that descriptively indicated the quality of laboratory or clinical practice: excellent (91% - 100%), good 76% - 90%), fair (66% - 75%) and insufficient (<66%). The cut-off value for good clinical practice was therefore ≥76%. The quality categories for individual sub-areas of services achieved by the assessed ART centres are shown graphically. For each sub-area from the checklists, the mean scores and minimum and maximum were calculated.

## Results

Interest in the ESHRE ARTCC programme among centres from different countries is shown in Table I. From September 2018 to December 1, 2021, a total of 63 clinics started an online application. More than half of them (38) were later excluded for various reasons: 11 were not from Europe (three from India, two from Morocco, and one each from Indonesia, Nigeria, Mexico, Northern Cyprus, Uzbekistan and Thailand), five were double submissions, 22 did not update the application for more than 1 year and did not reply to reminders. The remaining 25 were in various stages of application / certification as of 1st December 2021.

In the first half of 2019, two site visits were performed, and the first two ART centres were certified during the 35th ESHRE Annual Meeting, in 2019. Six more centres were assessed by live site visits (two) and virtual visits (four) between the second half of 2019 and 2021. By 1st December 2021, the evaluation process was completed for eight applicants in total, of which seven were certified while one centre did not reach the minimum score required for certification. The results of the evaluation of ART centres which completed the programme are shown in Fig. 2.

An average value of excellence (≥91%) was reached for "*Authorizations*" (97.4%) in Checklist-General services, for "*Laboratory cleaning*" (92.3%), "*Transportation of biological material*" (91.5%) and "*Consumables*" (91.6%) in Checklist-Laboratory services, and for "*Protective measures*" (91.5%), "*Psychology/Counselling*" (93.7%), and "Nursing" (92.8%) in Checklist-Clinical services.

Based on the average scores recorded, good practice status (≥76%) was not reached for "*Training / education*" in General services (68.2%) and "*Staffing and direction*" in Laboratory services (75.8%).

Among the problematic areas were those that in more than one-third of assessed centres did not achieve the status of excellent or good clinical practice and were classified as fair or insufficient. These included "*Training / education*" and "*Work organization and responsibility*" in General services, "*Staffing and direction*", "*Handling biological material*" and "*Quality management*" in Laboratory services and "*Staffing and direction*" and "*Possibility of surgery*" in Clinical services.

Individual comments and recommendations by inspectors are presented in Supplementary Tables SI-SIV. Only a summary of the most common recommendations is provided in this paper.

## Discussion

The ARTCC programme was not specifically advertised except at its launch during the 2018 ESHRE Annual Meeting. Nevertheless, in 3 years it sparked an unexpected interest in ART centres from as many as 20 European countries. Centres from other continents also tried to apply, although in this first stage access to the certification programme was restricted to centres located in Europe, as defined by ESHRE Internal Rules.

The ESHRE ARTCC represents an innovative and multidisciplinary approach for the assessment of good laboratory and clinical practice in ART centres for the benefit of both professionals and patients. Evaluation is focused on criteria that are commonly accepted as effective quality standards and performance indicators for ART within the reproductive medicine professionals.

Four checklists consisting of a total of 255 questions represent the core of the system. They were elaborated by experts following a meticulous review of EUTCDs, ESHRE guidelines and scientific literature on these subjects. For applicants, they provide a useful self-assessment tool. The complexity and comprehensiveness of ARTCC checklists ensures the value and reliability of this certification. It should be noted that this might also be the reason for the relatively large dropout rate or for the long time required by some centres to complete the application process. It is reasonable to assume that when filling in the checklists, some applicants may realize that some aspects of their clinical and laboratory activities do not comply with the standards required to obtain the certification.

Checklists also function as a reference point for inspectors to help them remember to verify compliance with all critical aspects and to allow an unbiased and coordinated assessment of general, laboratory and clinical practice. In addition, the objectivity of the assessment is ensured by analysis of data provided by the centres and by the constant supervision of the ARTCC Steering Committee.

To harmonise the work of inspectors, two preparatory courses were organized for them. Two refresher courses were also organized before the introduction of virtual visits to illustrate their functioning. This paper, describing all the inspectors' recommendations to date, provides useful learning material for regular inspectors' courses in the future.

The visit to the ART centre by the two inspectors is the key phase of the assessment process, as it aims to check whether the information provided in the checklists is correct. During the visit, both inspectors should observe closely all procedures performed at the centre and take the opportunity to clarify potential doubts arising from checklist assessment. Unfortunately, owing to the COVID-19 pandemic, the ARTCC Committee was forced to temporarily replace live site visits with virtual ones. This approach allowed the programme to continue. However, despite a fully functioning technical platform and a very well-designed assessment strategy, based on the experience of inspectors and ARTCC Steering Committee members, virtual visits were not fully comparable to a live visit in terms of effectiveness. A live visit gives inspectors a broader view and allows them to discover critical details, while a virtual visit is remotely targeted. For this reason, it was agreed that live site visits will be the standard and that they will resume at operating speed once the pandemic situation will allow doing so safely. In the future, virtual site visits will be restricted to any additional checks that are deemed necessary, or under specific circumstances requiring them.

Analysis of final reports has highlighted common critical areas of nonconformities in ART practice. This review paper highlights these areas to enable healthcare professionals to review if these nonconformities currently take place in their centres and to put corrective action in place. Inspectors will pay particular attention to these highlighted areas in the future.

*General services*

With reference to the Checklist-General Services (Supplementary Table SI), analysis of the recommendations allowed us to identify some non-compliances related to absence of agreements with other institutions in case of cessation of activities, laboratory air quality control, batch number traceability system and reporting of serious adverse reactions and events (SARE). These critical issues are otherwise covered by the EUTCD and were more commonly identified in non-EU countries. Furthermore, the absence of any documented training in reproductive medicine or embryology for clinicians holding the position of Laboratory Director or for clinical and laboratory staff was often observed. The need for documented training, verification of competencies and skills, and continuous professional education was among the most common comments from inspectors.

Since ESHRE provides educational activities and individual certifications for subspecialists in reproductive medicine, clinical embryologists, and reproductive nurses and midwives, participation in these certification programmes is the most common recommendation in this respect. While in some European countries reproductive medicine is already part of the residents' obstetrics and gynaecology training (Calhaz-Jorge et al., 2015), formal training and education in clinical embryology and ART laboratory practice continues to be a major problem as it is rarely covered in any undergraduate study curriculum, as shown in an ESHRE survey on this subject (Kovačič et al., 2015).

*Laboratory services*

Remarks about the Checklist-Laboratory Services (Supplementary Table SII) referred to deficiencies in manipulation and cryopreservation of infectious biological material and in the maintenance of constant physico-chemical and hygienic standards during the processing of reproductive cells and embryos.

General principles of good practice in IVF laboratories, such as recording relevant documentation (SOPs, lab forms, use of patient consent forms, third party agreements, etc.), set up of all types of traceability procedures, existence of hygienic standards and prevention of biological sample cross-contamination, quality control system of critical lab parameters in place, and risk management procedures described, are illustrated in detail both in ESHRE and World Health Organization guidelines (ESHRE Guideline Group on Good Practice in IVF Labs, 2016; WHO, 2021). Furthermore, these areas are strictly regulated the EUTCDs (European Parliament and the Council, 2004). Standards set in these documents should be widely accepted and implemented in centres in the EU. However, non-compliance with some EUTCD provisions (e.g. lack of contracts with another institution in case of cessation of activities; insufficient traceability of culture media and consumables by batch numbers; manipulation of infectious material; absence of SARE recording and reporting protocol) were detected exclusively in centres located outside of the EU.

It was concerning to find that in some countries, only a clinician can be a Laboratory Director, even with no evidence of laboratory experience and laboratory activity. ESHRE inspectors/embryologists also drew attention to shortcomings in the handling of biological material identified during checklist assessment and site visits, and they made recommendations for safer implementation of procedures such as ICSI (e.g. to process less oocytes per ICSI dish) and embryo assessment (e.g. the need for better control of temperature and culture dishes during embryo manipulation outside incubators). Laboratory quality management systems were generally relatively good in tracking physical parameters in incubators. However, several recommendations were made with reference to insufficient measures for contamination prevention.

Finally, it was reported that verification of laboratory KPIs described in the ESHRE paper (ESHRE Special Interest Group of Embryology and Alpha Scientists in Reproductive Medicine, 2017) is not frequently performed. In particular, internal quality assessment via verification of direct observation of procedural skills (DOPS) among laboratory staff (e.g. fertilization rate after ICSI per staff member) was rarely implemented.

*Clinical services*

While general and laboratory requirements are defined in detail in guidelines and directives, it was more difficult to evaluate objectively all relevant aspects of clinical checklists. While there are ESHRE guidelines on relevant subjects, treatment approaches may vary significantly based on individual practitioners' clinical evaluations and decisions.

Most common recommendations about Checklist-Clinical Services (Supplementary Table SIII) refer to the need for systematic implementation of a QM system (QMS) and individual clinician performance monitoring. Information on the potential risks related to specific ART techniques reported in consent forms was sometimes considered insufficient. Regarding reproductive surgery, it was frequently observed that centres did not have written agreements with other clinics/hospitals for referral of patients in need of surgical procedures in case the IVF Unit could not provide them. Other common recommendations were focused on a critical analysis of available techniques and on clinical criteria for their application.

Periodic verification of clinical KPIs (e.g., pregnancy rate per individual gynaecologist) is also infrequent. Its implementation should therefore be encouraged as a fundamental part of total QM.

*Treatments and clinical results*

Analysing and evaluating the tables with treatments and clinical outcomes turned out to be a difficult task, because a formal consensus among inspectors and ARTCC Steering Committee members on how to evaluate the extensive use of non-evidence-based techniques and or add-ons was not fully reached yet. This difficulty is mostly a result of the controversial literature on these subjects. In Supplementary Table SIV, for example, there are comments recommending to avoid overusing ICSI, preimplantation genetic testing for aneuploidies (PGT-A), freeze-all and other add-ons. At present, any recommendations on these aspects could only be based on clinical experience and on some publications describing the issue of widespread use of add-ons (Harper et al., 2012; Provoost et al., 2014; Repping, 2019; Zhang et al., 2020; Jans et al., 2020; Nardo and Chouliaras, 2020; Human Fertilisation & Embryology Authority, 2019, www.hfea.gov.uk/treatments/treatment-add-ons/). Clear statements issued by ESHRE Special

Interest Groups regarding such practices would therefore be very helpful to the ESHRE ARTCC programme.

Comments on the lack of practice in more sophisticated laboratory procedures owing to the insufficient number of procedures performed (less than 20, e.g. embryo biopsy, testicular biopsy and ICSI with surgically retrieved sperm) prevailed. Comments on the excessive use of add-ons (100% ICSI, PGT-A, assisted hatching, freeze-all) follow.

One of the main objectives of onsite visits was to verify the authenticity of clinical results provided. This evaluation was subject to a significant number of variables such as: availability of an ART cycle in-house database including clinical and laboratory data; availability of electronic medical records; real-time update of database including all started cycles; availability of cycle-by-cycle online reporting; possibility of tracking changes; external control of collected results; follow up > 90% of treatment outcomes; and data availability on deliveries and newborns. Verifying these indicators turned out to be more challenging in virtual site visits than in on-site ones, so evaluation of clinical results had to be based mostly on trust.

In addition, comparing clinical results with national EIM data turned out to be difficult owing to the interim reporting gap of approximately 4 years between the last available report and the results provided by ART centres (referring to the last year for which data on pregnancy outcomes were available). Furthermore, over the last 4 years, ART treatment approaches have changed considerably. For example, the freeze-all strategy seems to be more frequently used, making it more difficult to calculate pregnancy rates per cycle – which until recently was considered one of the main clinical KPIs. Inspectors reported that in many cases clinical outcomes per fresh cycle could not be objectively assessed because of non-inclusion of the number of started cycles or the frequent use of freeze-all cycles and postponed embryo transfers. For this reason, the ARTCC Steering Committee is currently preparing a new table to evaluate the effectiveness of ART techniques, based on laboratory and clinical KPIs as defined in ESHRE publications on KPIs (ESHRE Special Interest Group of Embryology and Alpha Scientists in Reproductive Medicine, 2017; ESHRE Clinic PI Working Group, 2021).

## Conclusion

In a relatively short period, the ARTCC programme has received great interest from centres in most European countries, even though it was not widely advertised, and also against a backdrop of the global epidemiological situation. This report was based on a limited number of inspection reports, but it was possible to identify recurring areas of MAR that often deviate from good laboratory and clinical practice. ARTCC procedures did not change since the launch of the programme, but this analysis will also be the basis for further improvements, notably with reference to the methods used for evaluating clinical outcomes. Performing this kind of analysis on a regular basis may not only contribute to continuous improvement of the ARTCC programme, but also provide useful inputs for the definition of the ESHRE educational strategy in terms of specific training needs, maintenance of good practice standards and harmonisation in the management of controversial issues in ART.

**Data availability**
All data are incorporated into the article and its online supplementary material.

**Acknowledgements**
The authors thank to all ESHRE ARTCC inspectors and ESHRE Central Office for assistance.

**Authors' roles**
B.K. and L.G.: substantial contributions to conception and design, drafting the article, analysis and interpretation of data; S.G., A.V., T.E., B.W., S.S.: acquisition of data, revising the article critically for important intellectual content; C.P., W.v.G.: acquisition of data, substantial contribution to technical and administrative part of certification; all authors: final approval of the version to be published and agreement to be accountable for all aspects of the work in ensuring that questions related to the accuracy or integrity of any part of the work are appropriately investigated and resolved.

**Funding**
The ARTCC programme was developed and funded by ESHRE, covering expenses associated with the meetings.

**Conflict of interest**
The Steering Committee members who are the authors of this paper did not receive payments for the completion of this study. The authors declare that they have no conflicts of interest. The inspectors were remunerated for their work with an honorarium.

## Figure legends

**Figure 1** Roadmap of ESHRE ART Centre Certification for good clinical practice.
**(A)** First certification. (**B)** Certification renewal. CO: Central Office

**Figure 2** The results of the evaluation of ART centres which completed the programme.
All data are percentage scores. OR: operating room

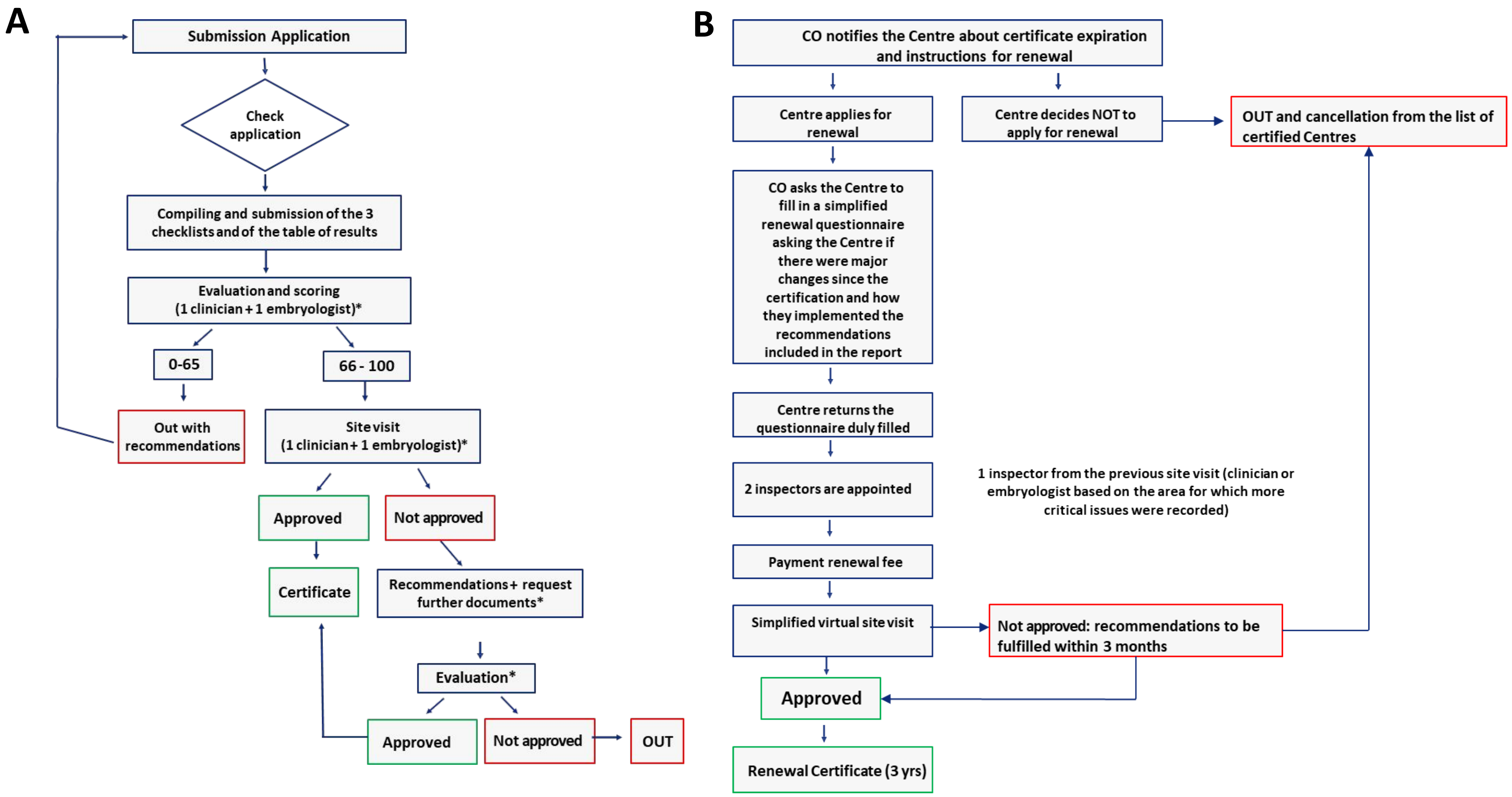

A
Submission Application
Check application
Compiling and submission of the 3 checklists and of the table of results
Evaluation and scoring (1 clinician + 1 embryologist)*
0-65
66 - 100
Out with recommendations
Site visit (1 clinician + 1 embryologist)*
Approved
Not approved
Certificate
Recommendations + request further documents*
Evaluation*
Approved
Not approved
OUT
B
CO notifies the Centre about certificate expiration and instructions for renewal
Centre applies for renewal
Centre decides NOT to apply for renewal
OUT and cancellation from the list of certified Centres
CO asks the Centre to fill in a simplified renewal questionnaire asking the Centre if there were major changes since the certification and how they implemented the recommendations included in the report
Centre returns the questionnaire duly filled
2 inspectors are appointed
1 inspector from the previous site visit (clinician or embryologist based on the area for which more critical issues were recorded)
Payment renewal fee
Simplified virtual site visit
Not approved: recommendations to be fulfilled within 3 months
Approved
Renewal Certificate (3 yrs)

**Figure 1**

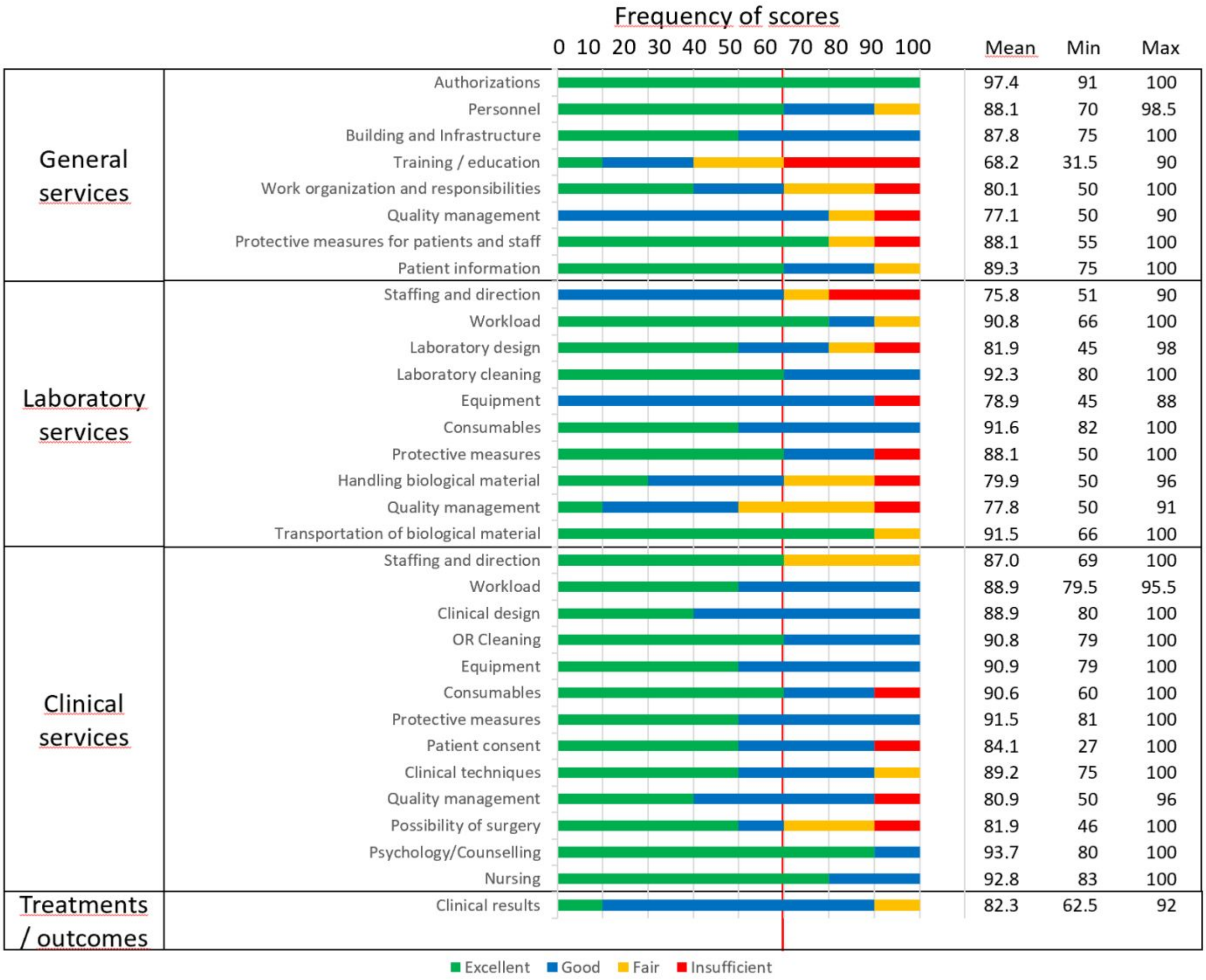

Frequency of scores
0 10 20 30 40 50 60 70 80 90 100
Mean Min Max
General services
Authorizations 97.4 91 100
Personnel 88.1 70 98.5
Building and Infrastructure 87.8 75 100
Training / education 68.2 31.5 90
Work organization and responsibilities 80.1 50 100
Quality management 77.1 50 90
Protective measures for patients and staff 88.1 55 100
Patient information 89.3 75 100
Laboratory services
Staffing and direction 75.8 51 90
Workload 90.8 66 100
Laboratory design 81.9 45 98
Laboratory cleaning 92.3 80 100
Equipment 78.9 45 88
Consumables 91.6 82 100
Protective measures 88.1 50 100
Handling biological material 79.9 50 96
Quality management 77.8 50 91
Transportation of biological material 91.5 66 100
Clinical services
Staffing and direction 87.0 69 100
Workload 88.9 79.5 95.5
Clinical design 88.9 80 100
OR Cleaning 90.8 79 100
Equipment 90.9 79 100
Consumables 90.6 60 100
Protective measures 91.5 81 100
Patient consent 84.1 27 100
Clinical techniques 89.2 75 100
Quality management 80.9 50 96
Possibility of surgery 81.9 46 100
Psychology/Counselling 93.7 80 100
Nursing 92.8 83 100
Treatments / outcomes
Clinical results 82.3 62.5 92
Excellent Good Fair Insufficient

**Figure 2**

**Table I** Data on ESHRE certification of good laboratory and clinical practice in ART centers on 1st December 2021 (European countries only).

| | Excluded owing to no progress in application after 1 year | Initial application (concept) | Submitted initial | Waiting for 1st payment | Approved for checklists | Submitted checklists | Waiting for 2nd payment | Approved for site visit | Reporting | Insufficient site visit score | Certified | Total |
|---|---|---|---|---|---|---|---|---|---|---|---|---|
| **Belgium** | | | | | | | | | | | **2** | **2** |
| **Bulgaria** | **1** | | | | | | | | | | | **1** |
| **Cyprus (Republic)** | **2** | | | | | | | | | | | **2** |
| **Czech Republic** | | | | | | | | | | | **1** | **1** |
| **France** | **1** | | | | | | | | | | **1** | **2** |
| **Georgia** | | | | | | **1** | | | | | | **1** |
| **Greece** | **1** | | | | | | | | | | | **1** |
| **Hungary** | | | | | | | | | | | **1** | **1** |
| **Italy** | **1** | | **1** | | | | | **1** | | | | **3** |
| **Moldova** | | | | | | **1*** | | | | | | **1** |
| **Poland** | **1** | **1** | | | **2** | | | | **1** | | **1** | **6** |
| **Portugal** | **1** | | | | | | | | | | | **1** |
| **Romania** | **1** | | **1** | | | | | | | | | **2** |
| **Russia** | | | | | | | | | | **1** | | **1** |
| **Spain** | **3** | | | | **1** | | | | | | | **4** |
| **Switzerland** | **1** | **1** | | | | | | | | | | **2** |
| **The Netherlands** | **2** | | | | | | | | | | **1** | **3** |
| **Turkey** | **3** | **1** | | | **2** | | | | **1** | | | **7** |
| **UK** | **2** | | | | | | | | | | | **2** |
| **Ukraine** | **2** | | | **1** | | | | | **1** | | | **4** |
| **TOTAL** | | | | | | | | | | | | **47**** |

*Resubmission following previous insufficient score

**TOTAL does not include excluded applications from non-European countries and double submissions

## Supplementary Material

hoac040_Supplementary_Data

