# Supplementary material for: ESHRE certification of ART centres for good laboratory and clinical practice
Source: Hum Reprod Open. 2022 Sep 14;2022(4):hoac040. doi: 10.1093/hropen/hoac040 (PMC9494398; doi:10.1093/hropen/hoac040)
Supplement: hoac040_Supplementary_Figure_S1 [file hoac040_supplementary_figure_s1.docx]

**
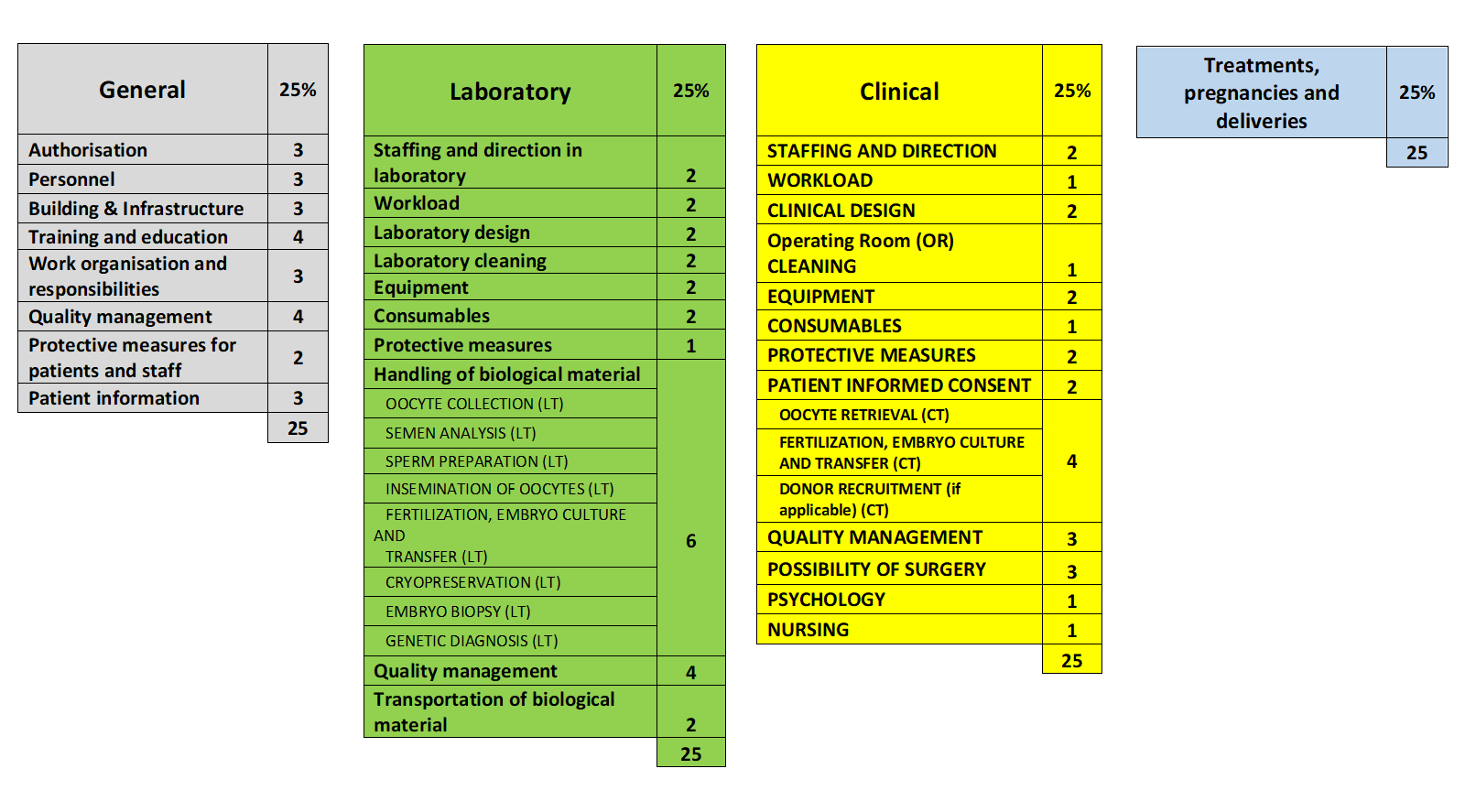
**

**Supplementary Figure S1** The weights of the individual domains from the four checklists, which together contributed 25% of the final score each.

LT: Laboratory task, CT: Clinical task
