# Supplementary material for: ESHRE certification of ART centres for good laboratory and clinical practice
Source: Hum Reprod Open. 2022 Sep 14;2022(4):hoac040. doi: 10.1093/hropen/hoac040 (PMC9494398; doi:10.1093/hropen/hoac040)
Supplement: hoac040_Supplementary_Figure_S2 [file hoac040_supplementary_figure_s2.docx]

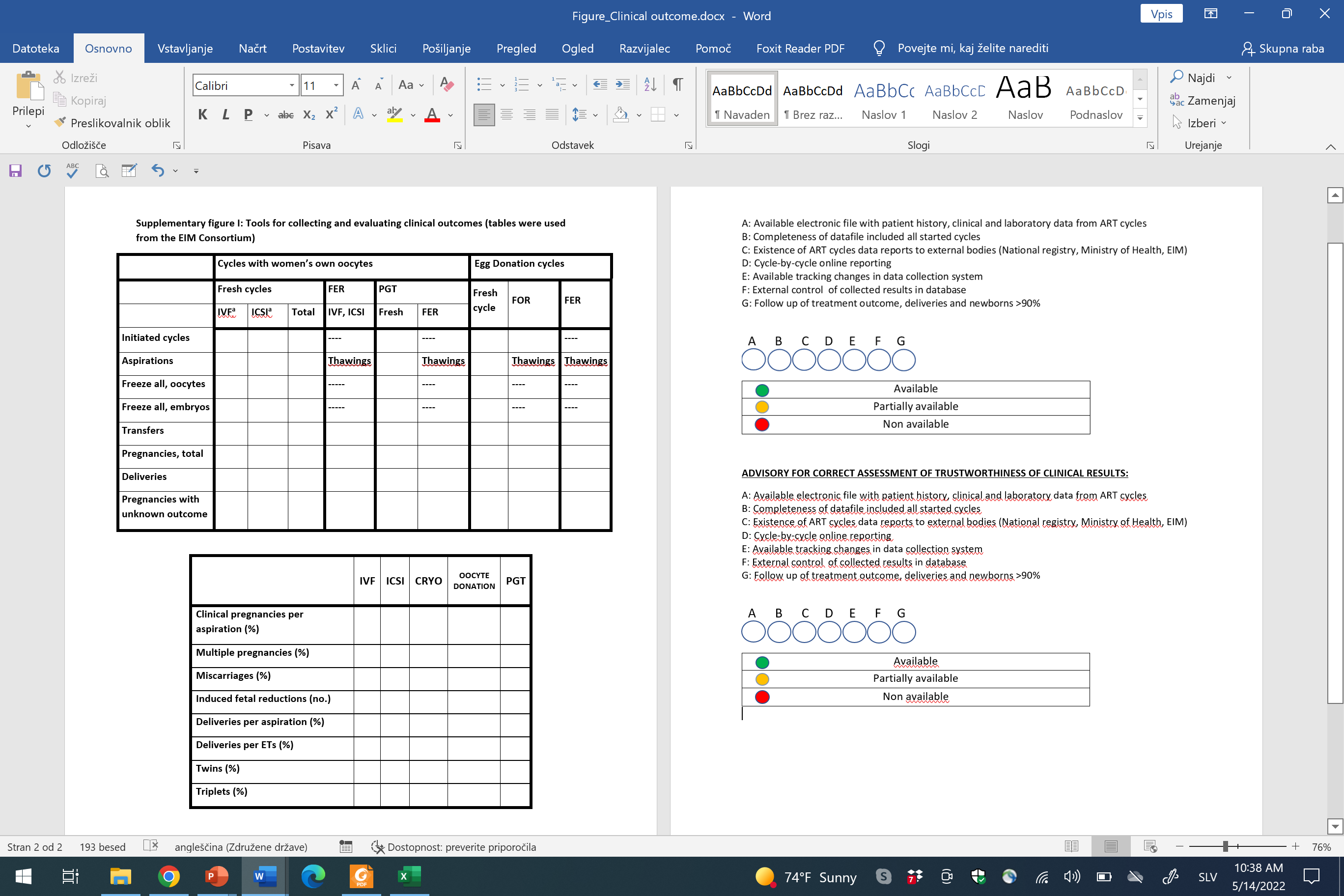

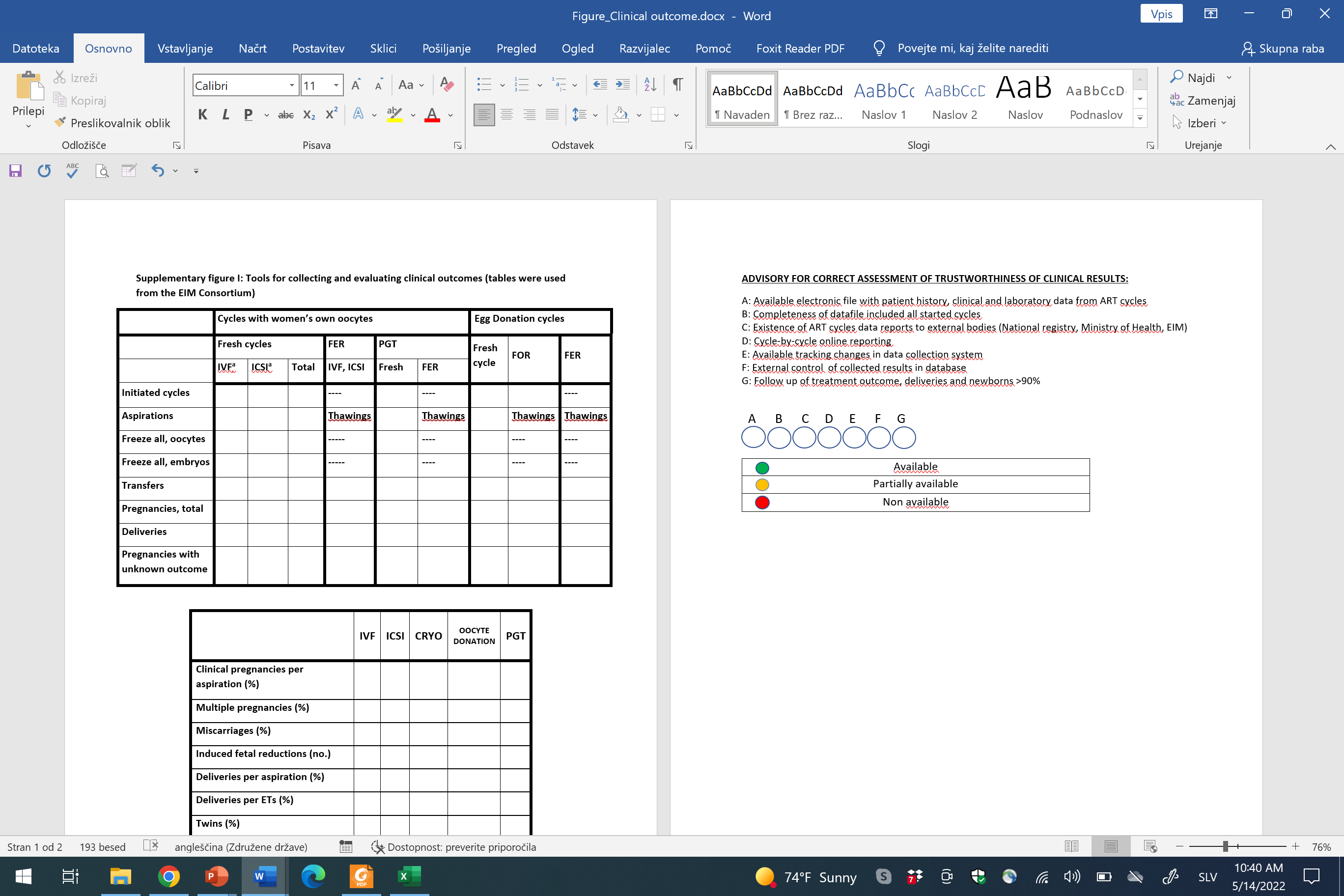


**Supplementary Figure S2** Tools for collecting and evaluating clinical outcomes (tables are from the EIM Consortium; traffic light system was developed to assess the trustworthiness in results).

EIM: The European IVF-Monitoring Consortium, FER: Replacement of frozen/vitrified embryos, FOR: Cycles with replacement of embryos derived from frozen/vitrified oocytes, PGT: preimplantation genetic testing, Cryo: cycles with embryo transfer of cryopreserved embryos, ET: embryo transfer
