# Supplementary material for: ESHRE certification of ART centres for good laboratory and clinical practice
Source: Hum Reprod Open. 2022 Sep 14;2022(4):hoac040. doi: 10.1093/hropen/hoac040 (PMC9494398; doi:10.1093/hropen/hoac040)
Supplement: hoac040_Supplementary_Table_SI [file hoac040_supplementary_table_si.docx]

**Supplementary Table SI** Major comments and recommendations about general services from certification reports.

| **Authorizations**   - Missing the contingency contract with another clinic to transfer cryopreserved material in case of cessation of center activity. |
| --- |
| **Personnel**   - No andrologists in the team. - Laboratory director is a clinician, without any documented experience with laboratory procedures. |
| **Building and Infrastructure**   - Insufficient laboratory space for safe workflow given the high number of cycles. - Sterile consumables are not unpacked from original cartoons outside the clean area. - Accesss to cryolab is not secured or protected by a low gas alarm. - Insufficient individual tracking of people entering the lab. - Background air of the andrology processing lab is not filtered and air quality is not controlled. |
| **Training / education**   - Laboratory staff and clinicians lack documents to verfiy their theoretical and practical knowledge in the field. - The staff members should be motivated to apply for ESHRE certification of individuals and to acquire documents by which they can prove their specialized education in the field of Reproductive Medicine / Clinical Embryology / Nursing. - No formal training programme (syllabus) in infertility treatment and ART procedures for clinicians and lab staff. - No defined minimum number of MAR/ART procedures to be performed under supervision. - Lack of documented logbook/datasheet identifying single steps of training with the final conclusion of training, date of end of training and signature of both participants (trainee and supervisor). - Competences and skills of clinicians and embryologists are not regularly verified with written protocol. - Protected educational time is not focussed on fellow’s area of interest and addressing syllabus requirements. - Education for trainee out of center is not formalized for missing parts like reproductive surgery or endocrinology (letter of intent with other center). - Too infrequent joint team meetings to evaluate treatment outcomes and journal club meetings. - Lack of organised educational/training meetings concerning updates in MAR for all staff members. - Participation in scientific meetings in country and abroad should be supported for clinicians, embryologists and nurses. - Lack of evidence of CPD/CME for embryologists, clinicians and nurses. |
| **Work organization and responsibilities**   - Lack of laboratory staff representative input into clinical decisions, particularly about oocyte insemination method. - Lack of permanent verification of the fulfillment of tasks by the head of laboratory. |
| **Quality management**   - Lack of: - regular document review. - SOPs for serious adverse reactions / event - monitoring clinicians’ performance - documentation of Covid19 management in SOPs or become part of the QM system. - policy for errors and incidents for clinical disciplines other than embryology - KPIs: - Not regularly checked and discussed with all staff members. - Do not incorporate collected data as part of IQC for the laboratory and clinic. Data are not evaluated to suffiicient frequency. - Head of the lab has not defined frequency for checking all KPIs, as per ESHRE »KPI paper«. - The clinical outcomes of the centre are not commented by any external authority. - Risk assessment in surgery does not exist. |
| **Protective measures for patients and staff**   - No gloves policy. - PCR testing of patients for Covid-19 before OPU, IUI, ET, regarding ESHRE guidelines is not in place. - Periodic PCR testing for Covid-19 of the team members is not in place. - The lack of reassessment the efficiency of the cleaning protocol of the lab/OR and reception areas for the COVID period. |
| **Patient information**   - No provision of detailed written information about the specific ART. - Staff name badges are missing and could help inform patients and other staff members of the person and role. |

MAR: Medically assisted reproduction, CPD: Continuing professional development, CME: Continuing medical education, SOP: Standard operating procedure, QM: Quality management, IQC: Internal quality control, KPI: Key performance indicators, OPU: Oocyte pick-up, ET: Embryo transfer, OR: Operating room
