# Supplementary material for: ESHRE certification of ART centres for good laboratory and clinical practice
Source: Hum Reprod Open. 2022 Sep 14;2022(4):hoac040. doi: 10.1093/hropen/hoac040 (PMC9494398; doi:10.1093/hropen/hoac040)
Supplement: hoac040_Supplementary_Table_SII [file hoac040_supplementary_table_sii.docx]

**Supplementary Table SII** Major comments and recommendations about laboratory services from certification reports.

| **Staffing and direction**   - The laboratory director is a clinician without any documents to demonstrate competence for this position. It is recommended to follow ESHRE curriculum and to apply for subspecialist examination for Reproductive Medicine or certification for Senior Clinical Embryologist. - ESHRE certification at least for the lab manager would be recommended - The ‘ESHRE certification” culture should be strongly encouraged for both embryologists and technicians. - to introduce and check the CPD in the clinic or implement some international programs for it, eg. ESHRE CPD system for main embryologist at least. - The training program, skills' evaluation and CPD program should be improved for all staff (clinicians, embryologists, nurses). |
| --- |
| **Workload**   - With approximately 1,500 treatments for five full‐time embryologists the human resource planning should be reconsidered. - The workload should reflect the number of staff to ensure there are enough staff to safely handle the number of patient cases. - Since a single ET session can cover 25 ETs with the same team, a break scheduled half way through the session could ensure concentration is optimal for each ET. |
| **Laboratory design**   - The lab is too small and the number of incubators and working places are not sufficient for the number of performed cycles. - Sperm laboratory air quality is not of sufficient quality for handling semen samples for treatments. - The location of some equipment could be reconsidered. The ICSI workstations are located in the Class II flowhoods. - It is strongly recommended to remove nitrogen (N2) cylinders from the embryology lab to room considered for gas supply. - Monitoring level of liquid N2 twice a week is a feasible option but for the future an external alarm system should be considered. - To use an acoustic or any other type of alarm when semen sample arrives to the laboratory. - Given the cooler air temperature in the OP rooms, the temperature changes experienced by oocytes and embryos should be re-evaluated. |
| **Laboratory cleaning**   - There is no documented evidence, tested internally or by an external company, to support claims of grade A in D air quality. |
| **Equipment**   - Laminar flow hood not used for processing sperm samples for MAR. - The sperm processing SOP states rotation speed in rpm, rather than g, despite use of different centrifuges. - One incubator is set inactive, but is considered to be ready for use. Not marked as “out of order”. - Lack of: - clarification for selection for use of two types of culture incuabator (with and without reduced oxygen (O2)). - KPIs specific to the different models of ICSI stations. - dedicated equipment (flow hood, incubator, centrifuge, cryotank) for processing and storage of cells and tissies from patients with infectious diseases - quarantine tank for cells and embryos if no infection screening is available at the time of freezing/vitrification. - connection of alarm for incubators and cryotanks to an autodialler for out of hours. - automatic change over for gas cylinders |
| **Consumables**   - Explore with the supplier of culture media for addition of logger during the shipment. - Lack of recording of batches of consumables. - An annual audit of traceability of consumables/media is recommended to ensure the current system of stock control is working. |
| **Protective measures**   - Personel protective equipment: - Gloves not used when processing positive or potential positive biospecimens. - Masks and gloves not used in accordance with ESHRE guidelines. - No documented risk assessment to support co-storage of BBV+ and BBV- gametes/embryos. - Consider methods to contain FF/blood and reduce the risk of spillage, rather than discard all samples into the large open containers. - Cryostorage room needs to have an O2 sensor. |
| **Handling biological material**   - Sperm preparations for treatments should be done in air grade A (laminar flow hood). - During follicle aspiration, the tube should be kept in the heating block at all times. - Follicular fluid from several follicles should not be collected in a flask. The use of tubes are recommended. - The IVF laboratory does not use standardized semen analysis methods - no sperm counting chamber. - Review the semen assessment process to ensure the correct number of sperm is assessed. - The sperm processing protocol should be adapted to the sperm’s parameters in terms of gradient layers and centrifugation timing. - Too many oocytes (>10) per ICSI dish. - Timings: - Cumulus oocyte complexes are kept out of incubator for long time at the time egg collection - The time for fertilization and embryo quality checking is not standardized according to ESHRE guidelines. - Timing of insemination (4h) is not consistent when higher numbers of cycles are performed on the same day. - Four‐eye‐witnessing should also be performed for the vitrification process. - Slow freezing is in use for early embryos, while blastocysts are vitrified, but the staff could not explain the advantage of such an approach. - The clinic has the permission and intention to treat infectious patients, however semen from seropositive patients is processed using a normal swim-up procedure, where density gradient is state of art. - Tips with filters should be used when handling media and especially biological material. |
| **Transportation of biological material**   - There is no SOP describing requirements for transportation of biological material and instructions about creation of single European code (SEC). |

MAR: Medically assisted reproduction, CPD: Continuing professional development, SOP: Standard operating procedure, KPI: Key performance indicators, ET: Embryo transfer, OR: Operating room, FF: Follicular fluid, BBV+: Positive test for blood borne viruses, BBV-: Negative test for blood borne viruses
