# Supplementary material for: ESHRE certification of ART centres for good laboratory and clinical practice
Source: Hum Reprod Open. 2022 Sep 14;2022(4):hoac040. doi: 10.1093/hropen/hoac040 (PMC9494398; doi:10.1093/hropen/hoac040)
Supplement: hoac040_Supplementary_Table_SIII [file hoac040_supplementary_table_siii.docx]

**Supplementary Table SIII** Major comments and recommendations about clinical services from certification reports.

| **Staffing and direction**   - Foreign fellows don’t speak internal language or poorly when recruited. This exposes the risk error in understanding internal documents/rules/procedure, write operative/consultation protocols. |
| --- |
| **Workload**   - The workload does not reflect the number of staff to ensure there are enough staff to safely handle the number of patients. |
| **Clinical design**   - N.A. |
| **OR Cleaning**   - An air purification system in the OR is missing in combination with the use of hair covers for all, shoe and clothes covers for the patients. |
| **Equipment**   - No laparoscopic tower. - For 5 specialist and described workload one ultrasound in the exam rooms is not enough. - For sterilization, all instruments rubbing is performed with one instead of a double filling system, and is not signed by the person who sterilized them, and the date, time and sterilization paper indicators are also missing. |
| **Consumables**   - A tip (oocyte pickup needle) to tip (embryo catheter) traceability is not ensured and all consumables that come into contact with gametes and embryos are not registered. |
| **Protective measures**   - Use of additional PPEs to only masks and gloves that are currently used for the protection of the staff and patients. - Testing of serology (HCV/HBV/HIV/VDRL test) and covid19 for all staff members according to ESHRE guidelines. - During pandemic era a more thorough cleaning protocol and microbiological testing is recommended . |
| **Patient consent**   - Detailed Informative document a Specific consent form for every different ART technique should be provided. - OHSS risk should be added to the informed consent. |
| **Clinical techniques**   - It is recommended to guarantee to all patients the same treatment conditions. Programme should be available also during weekends. - Consider the routine use of povidone for vaginal asepsia. |
| **Quality management**   - KPIs should be implemented in clinical practice to monitor clinical results. - KPIs does not incorporate documentation about direct observation of procedural skills. It is recommended to periodically evaluate pregnancy rates per clinician. |
| **Possibility of surgery**   - Number of surgical procedures are very low to maintain competency in the department, except for diagnostic hysteroscopy. - Low incidence of hysteroscopies performed can be discussed. - No written document of collaboration with other hospital for referral of patients for surgery. - Risk assessment in surgery may be improved: frequently, the one who operates is not the doctor who saw the patient and indicated surgery. |
| **Psychology/Counselling**   - Consider some intermediate time-point information regarding embryo progression between oocyte-pick-up and blastocyst transfer. |
| **Nursing**   - N.A. |

N.A.: Not available, OP: Operating room, KPI: Key performance indicators, OHSS: Ovarian hyperstimulation syndrom, PPE: Personal protective equipment, HCV: Hepatitis C virus, HBV: Hepatitis B virus, HIV: Human immunodeficiency virus, VDRL test: Venereal disease research laboratory test for syphilis
