# Supplementary material for: ESHRE certification of ART centres for good laboratory and clinical practice
Source: Hum Reprod Open. 2022 Sep 14;2022(4):hoac040. doi: 10.1093/hropen/hoac040 (PMC9494398; doi:10.1093/hropen/hoac040)
Supplement: hoac040_Supplementary_Table_IV [file hoac040_supplementary_table_iv.docx]

**Supplementary Table SIV** Major comments and recommendations about treatments from certification reports.

| **Treatments**   - Underuse of techniques: - IUI - IVF - Overuse of techniques: - ICSI - Assisted hatching - Freeze-all - SOP with indications for mix IVF+ICSI cycles is missing. - Insufficient experience in techniques: - oocyte vitrification because of a low number of cases. - embryo biopsy because a low number of PGT cycles are performed. - surgical sperm retrieval because of a low number of cases. - testicular sperm cryopreservation because of a low number of cases - Fine-needle biopsy cannot be the only method for surgically sperm retrieval. - Predominant HSG diagnostics instead HyCoSy - Reproductive surgery is not performed although center has Reproductive Medicine subspecialist training - Centre is certified for operative and diagnostic hysteroscopy. For laparoscopy, it should be clarified is there any hospital they could cooperate with. - Number of hysteroscopies performed annually should be considerably increased (10 is a too small number) if center is active in Reproductive Medicine education. - Number of surgical procedure are very low to maintain competency in the department, except for diagnostic hysteroscopy |
| --- |
| **Clinical outcomes**   - Cycle cancellation rate is too high. - Clinical pregnancy rate per aspiration is the same as delivery rate per ET. - Abortion rate is not reported. - Clinical pregnancy rate per aspiration and delivery rate per ET are below national average. - Twin rate is above EIM average. - Clinical outcome per fresh cycle can not be calculated because of too many freeze-all cycles. |
| **Verification of clinical results**   - Lack of availability of: - Electronic file with patient history, clinical and laboratory data from ART cycles - Tracking changes in data collection system - Cycle-by-cycle online reporting - Completeness of data file is insufficient; started cycles are not included. - No external control of collected results in database - Follow up of treatment outcome, deliveries and newborns is below 90%. |

SOP: Standard operating procedure, PGT: Preimplantation genetic testing, HSG: Hysterosalpingography, HyCoSy: Hysterosalpingo contrast sonography, EIM: The European IVF-Monitoring Consortium, ET: Embryo transfer
